# Diagnosis of growth hormone (GH) deficiency: comparison of pituitary stalk interruption syndrome and transient GH deficiency

**DOI:** 10.1186/1471-2431-9-29

**Published:** 2009-05-06

**Authors:** Murielle Louvel, Mariana Marcu, Christine Trivin, Jean-Claude Souberbielle, Raja Brauner

**Affiliations:** 1 Université Paris Descartes and AP-HP, Hôpital Bicêtre, Unité d'Endocrinologie Pédiatrique, Le Kremlin Bicêtre, 94275, France; 2 AP-HP, Hôpital Necker-Enfants Malades, Service d'Explorations Fonctionnelles, Paris, 75743, France

## Abstract

**Background:**

Most patients with childhood non-organic growth hormone (GH) deficiency (GHD) produce a normal GH peak as young adults. Our objectives were to better define this transient GHD and evaluate the factors influencing the growth response of patients with pituitary stalk interruption syndrome (PSIS).

**Methods:**

We studied 72 prepubertal patients with a GH peak < 6.7 ng/ml after 2 stimulation tests, treated with 0.2 mg GH/kg/w for at least 3 years. Group 1 (n = 53, 4.7 ± 4.0 years) had PSIS and Group 2 (n = 19, 9.2 ± 3.0 years) had transient GHD and normal pituitary.

**Results:**

At diagnosis, 64% of Group 1 and one Group 2 were < 5 years old. The growth rate of 59% Group 1 and two Group 2 patients was ≤ -2 SDS. The GH peak of 64% Group 1 patients and no Group 2 patients was < 3 ng/ml. The plasma insulin-like growth factor-1 of all Group 1 and all but one Group 2 patients was ≤ -2 z scores.

During the first year of GH treatment, the growth rate was ≥ 2 SDS in 81% Group 1 and 37% Group 2 patients. In Group 1, it was negatively correlated with the GH peak before treatment (P < 0.03), and with the difference between the target and adult heights (P < 0.01).

The height gain SDSs between diagnosis and adult height were 1.7 ± 1.2 in Group 1 (n = 30) and 1.08 ± 0.8 in Group 2 (n = 12, P = 0.05).

**Conclusion:**

The factors of the growth response to GH treatment should be analysed separately for each population: with and without PSIS or other markers.

## Background

Growth hormone (GH) deficiency (GHD) due to damage to the hypothalamic-pituitary region by lesions, surgery and/or radiation is said to be "organic". In other circumstances, GHD is readily diagnosed when magnetic resonance imaging (MRI) shows pituitary stalk interruption syndrome (PSIS) [1] and/or if there is microphallus, hypoglycemia, other hypothalamic-pituitary deficiencies, or genetic causes. GHD is difficult to diagnose when none of these conditions apply, the GHD is then said to be "idiopathic".

GHD is diagnosed by the height of the GH peak after pharmacological stimulation. The value of these tests has been questioned [2] because they are expensive, labor intensive, occasionally risky, and their results are not very reproducible. The majority of patients who have no markers of certain GHD produce a normal GH peak response to these tests when evaluated as young adults [3]. The growth rate and plasma insulin-like growth factor (IGF) 1 concentration of these patients with "transient" GHD are frequently low for their chronological age at diagnosis [4]. The question is whether the transient GHD reflects a true transient decrease in GH secretion or an insufficient response to the stimulation test, as occurs in many short or normal height, non-GHD patients [5]. The transient GHD may be partly responsible for the variability of the growth response to GH treatment.

We compared the characteristics at diagnosis, growth during the three first years of GH treatment, and adult height and concomitant GH evaluation of patients with GHD and PSIS to patients with transient GHD. Our first objective was to analyze the characteristics of transient GHD and compare them to that of a permanent GHD group, that with PSIS; our second objective was to evaluate the factors influencing the growth response of patients with PSIS.

## Methods

### Patients

The subjects of this retrospective longitudinal study were 72 consecutive prepubertal patients with GHD first seen by one of us (R. Brauner) in a university pediatric hospital. They were first seen from birth to 17 years, and treated with GH for at least 3 years.

Group 1 included 53 patients with GHD and PSIS. Fifteen other patients seen during the same period were not included because the PSIS was associated with malformative syndrome, which can modify the parameters studied (Fanconi anemia n = 6, Blackfan-Diamond anemia n = 1), or because their GH treatment was initiated elsewhere (n = 8).

Group 2 included 19 patients with transient GHD, initially diagnosed as having isolated idiopathic GHD because of a low GH peak after two stimulation tests and a normal pituitary on MRI. They were treated with GH and produced a normal GH peak after a third stimulation test. None of them had intrauterine growth retardation, metabolic disease or malformative syndrome.

The other patients treated during the same period for idiopathic GHD without PSIS or a demonstrated transient character were not included.

### Protocol

Informed consent for the evaluations and treatments was obtained from the children's parents. The Ethical Review Committee (Comité de Protection des Personnes Ile de France III) stated that "this research was found to conform to generally accepted scientific principles and research ethical standards and to be in conformity with the laws and regulations of the country in which the research experiment was performed".

The criterion for diagnosing GHD was a GH peak response of less than 6.7 ng/ml after two pharmacological stimulation tests, excluding the GH-releasing hormone test.

The first evaluation was performed in a single morning after an overnight fast, and included measurements of height and weight, bone age (except in those aged less than one year), a pharmacological stimulation test of GH secretion, and checks to exclude other causes of short stature. As the GH peak at the first test was subnormal, a second test using another stimulus was performed. As this second test also showed a subnormal GH peak, the program included MRI to exclude organic intracranial lesions and to look for PSIS, and evaluation of the other pituitary functions.

The replacement treatment for Group 1 patients included thyroxin (3–5 μg/kg/day) for 29 of them with thyroid stimulating hormone (TSH) deficiency, and hydrocortisone (6–17 mg/m2/day) for 23 of them with adrenocorticotropin deficiency. Thirty six patients reached pubertal age. The 15 of them who had no pubertal development despite being of pubertal chronological and bone ages underwent a gonadotropin releasing hormone stimulation test. It showed gonadotropin deficiency; the girls were given oral ethinyl estradiol (2 μg/day) from the age of about 12 years, and the boys were given testosterone heptylate (25 mg i.m. every 14 days) from around 13–14 years. These doses were continued during the growing period and increased to the adult dose when growth had ceased.

The patients on replacement treatment were seen every 3 (younger than 2 years) or 6 months to measure their height, weight and to adjust the GH, thyroxin and hydrocortisone doses for their weight, clinical features and, if necessary, plasma free thyroxin concentrations.

GH treatment was stopped when the height increase over the six previous months was less than 1 cm with a bone age greater than 15 years (boys) or 14 years (girls). Group 1 patients were treated for 9.9 years and Group 2 patients for 5.4 years (P < 0.0001), but the GH doses were similar (0.2 and 0.19 mg/kg/w). A third stimulation test was performed on 25 Group 1 patients and all Group 2 patients at least one month after the end of GH treatment. Adult height was reached by 30 Group 1 and 12 Group 2 patients.

### Methods

Height was measured twice with a Harpenden stadiometer. The height, growth rate and body mass index (BMI, weight in kg/height in m^2^) are expressed as standard deviation scores (SDS) for chronological age [6,7]. The growth rate before treatment of 6 patients aged less than one year at diagnosis was not calculated. The growth rates of the 4 patients of pubertal age but prepubertal stage were compared to the prepubertal values. Target height was calculated from parental heights [8] except for 4 adopted patients. Bone age was assessed by R. Brauner [9].

The results of GH treatment are expressed as the SDSs of the growth rates each year and the height changes each year and over 3 years of treatment, and of the adult height. They were compared in each group to the data at the start of GH treatment: chronological and bone ages and their difference, height, difference from target height, growth rate, BMI, GH peak, IGF-1 and with GH dose. The growth rate was also compared to the change in BMI during the same period and to the growth rate the previous year. In the Group 1, the patients with isolated GHD were compared to those having additional deficiencies, substituted with thyroxin and hydrocortisone. None of them were given sex steroids during the first three years of treatment.

GH secretion was assessed at diagnosis by the sequential arginine-insulin test and the ornithine test; a glucagon test was used for patients weighing less than 10 kg and those who were hypoglycemic. As the mean GH peak heights for the first and second pharmacological stimulation tests performed before GH treatment in each group were not different and as these evaluations were very similar, the larger GH peak and the concomitant plasma IGF-1 values were used for analysis. The third test used stimulation by arginine-insulin or glucagon.

GH was measured over the years using several different immunoassays calibrated against different reference preparations. The GH peaks were therefore recalculated to express them in ng/ml of the international reference standard 98/574 (recombinant 22 kDa GH, 1 μg = 3 mU), as requested in a recent consensus on GH assays [10]. We used the DiaSorin RIA competive assay (DiaSorin, Sallugia, Italy), calibrated against the 66/217 reference (1 μg = 2 mIU), the Immunotech IRMA kit (Immunotech, Marseille, France), calibrated against the 80/505 international standard (1 μg = 2.6 mIU), and most recently the Beckman-Coulter Access automated immunochemilumiscent assay (Beckman-Coulter, Chaska, Minnesota), now calibrated against the new 98/574 reference preparation (recombinant hGH, 1 μg = 3 mIU). As the results of these 3 assays were very similar when expressed in mU/l, we multiplied the results in μg/l (= ng/ml) obtained with the DiaSorin and the Immunotech assays by 2 and 2.6 respectively and then, for all the assays, divided the calculated results in mIU/l by 3.

IGF-1 was measured by immunoassay (IGF-1-RIACT, Cis Bio, Gif sur Yvette, France) and expressed in Zscore (zs) according to chronological age [11]. The control group at the first evaluation included normal prepubertal children, and at the third evaluation it included 31 adolescents aged 14–16 years and 30 young adults aged 17–20 years of normal height and weight and spontaneous pubertal development.

Other pituitary functions were evaluated by basal blood cortisol at 08.00 h, thyroxin, prolactin, and the TSH response to thyrotropin releasing hormone; the plasma and urinary osmolalities after water deprivation for 12-h were normal in all patients.

Data are expressed as means ± SD. Groups were compared with a Mann-Whitney U test. Correlations were analyzed using Spearman's test.

## Results

### 1. Characteristics at diagnosis (Table 1 and Figure 1)

**Figure 1:**
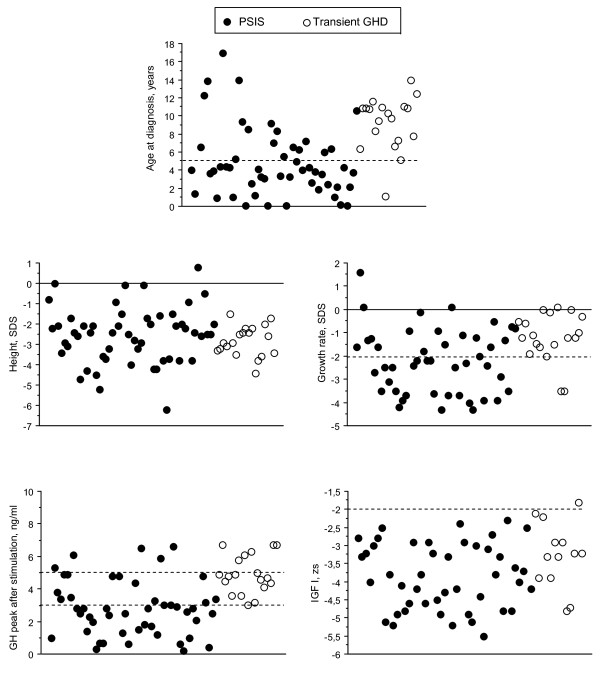
**Characteristics at diagnosis**.

**Table 1:** Patient characteristics

Group	N (boys/girls)	Age years (a)	Height SDS	Target height SDS (b)	Growth rate SDS (c)	BMI SDS	GH peak ng/ml (a)	IGF-1 zs
1. GHD with PSIS	53 (31/22)	4.7 ± 4.0 [0.02;17]	-2.5 ± 1.4 [-6.2; 0.8]	-0.2 ± 0.8 [-1.7;1.5]	-2.2 ± 1.4 [-4.3;1.6]	0.19 ± 1.7 [-2.4;6.7]	2.6 ± 1.8 0.0;6.6	-3.8 ± 0.9 -5.5;-2.3

2. Transient GHD	19 (9/10)	9.2 ± 3.0 [1.1;14.0]	-2.8 ± 0.8 [-4.4;-1.5]	-0.8 ± 0.9 [-2.6;0.9]	-1.2 ± 1.0 [-3.5;0.1]	0.32 ± 1.4 [-1.9;3.5]	4.9 ± 1.2 3.0;6.7	-3.2 ± 0.9 -4.8;-1.8

means ± SD [ranges]

Group 1 compared to Group 2: (a) P < 0.0001; (b) P < 0.02; (c) < 0.003

Among the 29 Group 1 patients with other hypothalamic-pituitary deficiencies, 4 had only a TSH deficiency, 10 had TSH and adrenocorticotropin hormone deficiencies, 2 had TSH and gonadotropin deficiencies, and 13 had a complete anterior pituitary deficiency.

Group 1: one girl had a paternal aunt with PSIS. Microphallus was present in 11/31 (35.5%) boys and cryptorchidism in 6/31 (20%) boys; 24/53 (45.3%) patients suffered from hypoglycemia and 29 (54.7%) had other hypothalamic-pituitary deficiencies. Among these, 4 had only a TSH deficiency, 10 had TSH and adrenocorticotropin hormone deficiencies, 2 had TSH and gonadotropin deficiencies, and 13 had a complete anterior pituitary deficiency.

Group 2: four of them had anterior pituitary hypoplasia (height < 4 mm, – 2 SD for chronological age) [1]

The sex ratio, height and weight at birth, height, difference between target and actual heights, BMI, plasma IGF-1 and difference between chronological and bone ages were similar in the two groups, but the patients with PSIS were significantly younger at diagnosis, and had greater target heights, slower growth rates and lower GH peaks than those with transient GHD.

Thus the age at diagnosis of GHD was less than one year in 6 Group 1 patients (including 5 aged less than one month), but in no Group 2 patients. It was less than 5 years in 34 (64%) Group 1 patients and in one Group 2 patient.

The growth rate was below the mean in 93.5% of the Group 1 patients and in 84.2% of the Group 2 patients. The 3 Group 1 patients with a growth rate above the mean included one born prematurely who underwent catch-up growth and another with a BMI of 6.2 SDS. The growth rate was ≤ -2 SDS in 59% of Group 1 patients and in two Group 2 patients.

The GH peak was below 3 ng/ml in 64% of Group 1 patients, but in no Group 2 patient. The corresponding percentages for a GH peak below 5 ng/ml were 90.4 and 63.1. The plasma IGF-1 was ≤ -2 zs in all Group 1 patients and in all but one (-1.8) Group 2 patients.

### 2. Growth rates on treatment (Table 2, Figures 2 and 3)

**Figure 2:**
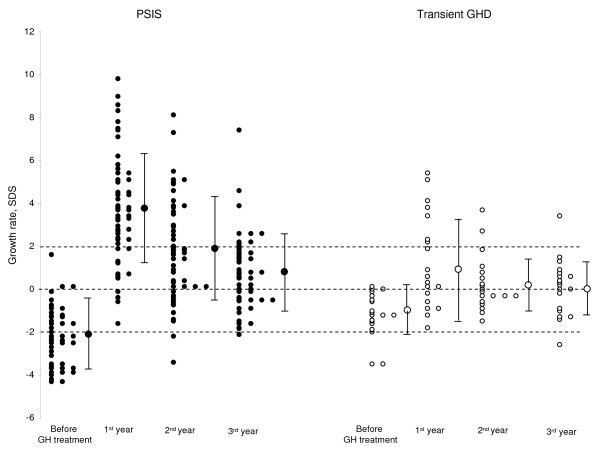
**Growth rates before and during the first three years of GH treatment**.

**Figure 3:**
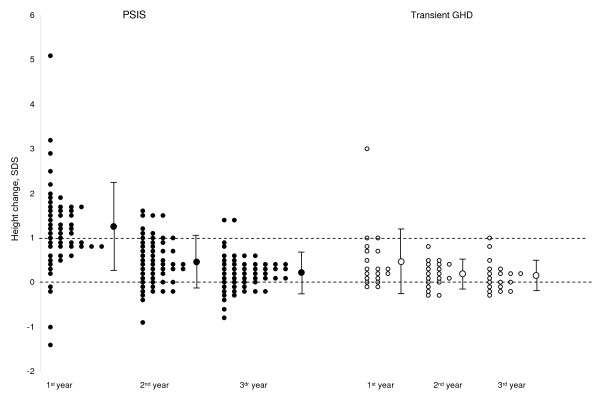
**Annual changes in height during the first three years of GH treatment**.

**Table 2:** The growth responses (SDS) of patients with PSIS or transient GHD to GH treatment

**SDS**	**PSIS**	**Transient GHD**	P
	(n = 53)	(n = 19)	
**First year**			
Growth rate	3.8 ± 2.6	1.3 ± 2.2	0.0007
Height change	1.2 ± 1.0	0.48 ± 0.7	<0.0001
**Second year**			
Growth rate	1.8 ± 2.4	0.26 ± 1.3	0.006
Height change	0.47 ± 0.5	0.18 ± 0.3	<0.03
**Third year**			
Growth rate	0.77 ± 1.7	0.05 ± 1.3	NS
Height change	0.2 ± 0.4	0.14 ± 0.3	NS
			
**Height change over three years**	1.9 ± 1.1	0.8 ± 1.0	0.0002
			

	(n = 30)	(n = 12)	
**Adult height**	-1.0 ± 0.9	-1.8 ± 0.8	<0.02
**Height change from diagnosis**	1.7 ± 1.2	1.08 ± 0.8	0.05
			

Mean ± SD			

The growth rates and the height changes of Group 1 patients were significantly greater than those of Group 2 patients during the first and second years of treatment, but not during the third. The height changes over three years were also greater in Group 1 than in Group 2. In Group 1, the growth rates each year and over three years were similar in the patients with isolated GHD to those having additional deficiencies.

The growth rate during the first year was ≥ 2 SDS in 81% of Group 1 patients and in 36.8% of Group 2 patients. The 9 Group 1 patients with growth rate < 2 SDS included three who were the oldest in the study. Their GHD was diagnosed between 14 and 17 years; their growth rates were compared to prepubertal values (see Methods). The 6 others, including 3 neonates, had hypoglycemia and adrenocorticotropin hormone deficiency and were treated with hydrocortisone (10–15 mg/m^2^/day). All had TSH deficiency and were treated with thyroxin (4 to 5 μg/kg/day).

The height change during the first year was ≥ 1 SDS in 62.5% of the Group 1 patients and in one Group 2 patient.

### 3. Evaluation at the end of growth and adult height

The adult height SDSs were -1 ± 0.9 in Group 1 and -1.8 ± 0.8 in Group 2 (P<0.02, Table 2): 171.5 cm for the boys and 155.8 cm for the girls in Group 1, and 162.7 (boys) and 155 cm (girls) in Group 2.

The GH peaks were 2.3 ± 2.9 ng/ml for the Group 1 and 14.7 ± 11.4 ng/ml for the Group 2 patients (P < 0.0001). The corresponding plasma IGF-1 concentrations were -3.6 ± 1.2 and -1.4 ± 1.3 zs (P < 0.0008). These concentrations were < -2 zs in all Group 1 patients and in 3/9 of the Group 2 patients. They were positively correlated with the GH peak for the whole population (P = 0.0002), but not within each group.

### 4. Correlation

Group 1: the growth rate during the first year of treatment was positively correlated with the BMI (P < 0.007) and negatively with the GH peak before treatment (P < 0.03). These correlations persisted when the 6 patients aged less than one year at diagnosis were excluded. The adult height SDS was positively correlated with the growth rate during the second year of treatment (P < 0.03) and with the target height (P < 0.01). The height gain between diagnosis and the adult height (1.7 ± 1.2 SDS) was negatively correlated with the height (P < 0.007) and positively correlated with the difference between the chronological and bone ages (P = 0.01) before treatment. The difference between the target and adult heights (0.7 ± 0.8 SDS) was negatively correlated with the growth rates during the first and third years (P < 0.01 for both).

Group 2: the growth rates and the height change over 3 years were not correlated with the parameters before treatment. The adult height SDS was positively correlated with the target height (P < 0.03).

## Discussion

This study shows that the patients with transient GHD have features that differ from those of patients with PSIS. In these, the growth rate during the first year on GH is negatively correlated with the GH peak before treatment, quantifying the degree of GHD, and with the difference between the target and adult heights. The young patients treated with hydrocortisone for symptomatic hypoglycemia had a lower growth response.

### 1. Characteristics of transient GHD

It was difficult to decide whether the Group 2 patients had a true transient decrease in their GH secretion or simply an insufficient response to the stimulation test. The majority had a growth rate below the mean and all but one had a plasma IGF-1 concentration < -2 zs at diagnosis. Their transient GHD is not explained by their BMIs being different from those of the PSIS patients, or by pubertal delay. One third of them had a growth rate of ≥ 2 SDS on GH treatment. And while their GH peak at the end of treatment was normal, 3/9 of them still had an IGF-1 of < -2 zs. Their growth in response to GH treatment was not correlated with their characteristics before treatment; however, this might be because their number is smaller than the Group 1 patients, while their different characteristics before treatment may have contributed to their lower growth response. The only correlation was between their adult and target heights. Their height gain SDSs between diagnosis and the adult height were lower than those of the patients with PSIS, but the difference is not significant.

We used logical data analysis, a combinatorial method that produces rules characterististic of subsets of either positive (patients with PSIS) or negative (patients with short stature and normal GH peak) patients [12]. We find that 74% of the patients with transient GHD were "in between" the positive and negative patients, while 26% were classified as negative.

Loche et al [13] found a normal GH peak in 28/33 patients having such characteristics, at a third evaluation performed after 1 to 6 months without GH treatment.

### 2. Factors influencing the growth response of PSIS patients

The published analyses of the factors influencing the growth response of GHD patients to GH treatment have probably been hampered by the inclusion of patients with transient GHD. Only three studies analysed patients with congenital hypothalamic-pituitary MRI abnormalities separately [14-16]. Zenaty et al [15] found the mean height gain of 32 patients with MRI abnormalities to be (2.2 SDS) after 3 years on 0.18 mg GH/kg/w, significantly greater than that of 37 patients without such abnormalities (1.6 SDS). Our results for the 47 patients with PSIS are similar, after exclusion of the 6 aged less than one year. Coutant et al [14] found that 15 patients with MRI abnormalities were significantly younger and shorter at diagnosis than 48 patients without such abnormalities; their height gain between diagnosis and the adult height on 0.15 mg GH/kg/w was greater (2.7 SDS) than that of patients without abnormalities (1.3 SDS) and their adult height was greater (-1.1 SDS compared to -1.7 SDS). When the patients without MRI abnormalities were retested, 22/35 of them had a GH peak of ≥ 10 ng/ml. We found similar adult heights, but the height gain for the PSIS patients was less (1.7 SDS), probably because our patients were younger and taller at diagnosis. This gain was negatively correlated with their height and positively correlated with the difference between the chronological and bone ages before treatment. Maghnie et al [16] reported the adult heights of 88 patients with permanent GHD; 73 of them had MRI abnormalities and were treated with 0.2 mg GH/kg/w. The heights of those with spontaneous and induced puberty were similar and the difference between the target and adult heights was around 0.4 SDS, while we found 0.7 SDS.

The growth during the first year of GH treatment has been reported to predict the adult height [17-19]. We find that the growth rate during the first year on GH is negatively correlated with the difference between the target and adult heights and with the GH peak before treatment, as did Cole et al [20]. They found that the strongest predictor of the first year growth response, adjusting for age, height, weight, target height and injection frequency, was the GH test result, suggesting that the growth response is the gold standard for quantifying the degree of GHD. Van den Broeck et al [21] showed that patients with severe GHD (a GH peak < 5 ng/ml) responded better to treatment, but that the responses of children with partial GHD and those with idiopathic short stature were similar. Wilson et al [22] found that all but the very lowest GH peaks were poor predictors of subsequent growth. Bright et al [23] reported that GH stimulation tests correctly identified 64% of the GH treatment outcomes: the increase in height during the first year was greater than 0.5 SDS in 56% of the patients with a GH peak <10 ng/ml, while it was less than 0.5 SDS in 8% of those with a GH peak > 10 ng/ml. Our data show that patients aged less than one year may have an insufficient growth response despite PSIS, because severe hypoglycemia with associated adrenocorticotropin deficiency may lead to the use of slightly high hydrocortisone replacement doses.

## Conclusion

Our current policy for those patients who have a low GH response to a stimulation test and normal MRI is to evaluate the clinical efficacy of GH therapy during the first two years. If their growth rate does not increase significantly, we propose a third evaluation of their GH secretion, after interrupting GH treatment for at least one month.

The factors influencing the growth response to GH treatment and the associated models should be analysed separately for each of the populations: with and without PSIS or other marker.

## Abbreviations

BMI: body mass index; GH: growth hormone; GHD: growth hormone deficiency; IGF: insulin-like growth factor; MRI: magnetic resonance imaging; PSIS: pituitary stalk interruption syndrome; SDS: standard deviation score; TSH: thyroid stimulating hormone; zs: Zscore

## Competing interests

The authors declare that they have no competing interests.

## Authors' contributions

ML and MM contributed to the project design, data acquisition and data analysis. CT and JCS carried out the immunoassays and the statistical analyses. RB directed the work and prepared the manuscript. All the authors have given final approval of the version to be published.

## Pre-publication history

The pre-publication history for this paper can be accessed here:


